# Bluetongue Virus Serotype 3 Follow‐Up of the 2024 Outbreak in Two Belgian Zoos

**DOI:** 10.1155/tbed/5805846

**Published:** 2026-04-18

**Authors:** Jonas Spruyt, Laurens Van Mulders, Ilse De Leeuw, Nick De Regge, Barbara Beci, Francis Vercammen

**Affiliations:** ^1^ Centre for Research and Conservation, Royal Zoological Society of Antwerp, Antwerp, Belgium; ^2^ Operational Direction Infectious Diseases in Animals, Unit of Exotic and Vector-Borne Diseases, Sciensano, Brussels, Belgium, sciensano.be; ^3^ Department of Internal Medicine, Reproduction and Population Medicine, Faculty of Veterinary Medicine, Ghent University, Merelbeke, East Flanders, Belgium, ugent.be

## Abstract

In summer 2024, Western Europe experienced extensive outbreaks of bluetongue virus Serotype 3 (BTV‐3), a Culicoides‐borne orbivirus. Clinical disease was first detected in August 2024 in one of two Belgian zoos, predominantly affecting European bison (*Bison bonasus*) and American bison (*Bison bison*). These species showed high morbidity, two fatalities, and severe multisystemic lesions at necropsy. This outbreak coincided with reemergence of BTV‐8 and increasing reports of emerging BTV‐12 and epizootic hemorrhagic disease virus (EHDV) circulation in Europe. Following the outbreak, samples from 116 animals representing 33 species were analyzed across two Belgian zoos (urban and rural), spanning a 20‐year period (2005–2025). Species belonged to Artiodactyla, Perissodactyla, Proboscidea, Rodentia, Diprodontia, and Carnivora. Archived sera predating 2024 (retrospective), samples collected during the 2024 outbreak (outbreak), and follow‐up samples obtained through June 2025 (follow‐up) were tested at the Belgian National Reference Laboratory (NRL). Antibodies were screened using a pan‐BTV ELISA, and ELISA‐positive samples were further analyzed by virus neutralization tests (VNTs) to differentiate BTV‐3 and BTV‐8. Viral RNA detection was performed using RT‐qPCR assays targeting pan‐BTV, BTV‐3, BTV‐12, and EHDV. Serological reactivity was confined to Artiodactyla and Perissodactyla. In total, 46 ruminants (67% of tested ruminants) and one greater one‐horned (GOH) rhinoceros (*Rhinoceros unicornis*) were ELISA positive. VNT confirmed BTV‐3 infection in 62% and BTV‐8 in 9% of seropositive ruminants. Viral RNA was detected in whole blood from 28 individuals, representing 42% of the tested ruminants. Marked epidemiological differences were observed between zoos, with limited detection in the urban collection and widespread infection in the rural collection, likely reflecting differences in vector exposure. No evidence of BTV‐12 or EHDV was detected. This study provides comprehensive documentation of BTV‐3 in zoo‐housed species, integrating clinical, pathological, and longitudinal surveillance data. It underscores pronounced interspecies variability in susceptibility and immune response, providing critical insights for outbreak preparedness.

## 1. Introduction

Bluetongue virus (BTV) is a member of the family Reoviridae, genus *Orbivirus*, and the causative agent of bluetongue disease in domestic and wild ruminants [1]. The virus is nonenveloped and has a genome of 10 linear double‐stranded RNA segments [2]. At least 36 serotypes have been identified. Serotypes 1–24 are considered “typical,” transmitted primarily by *Culicoides* biting midges, while Serotypes 25–36 are regarded as “atypical,” with evidence of direct animal‐to‐animal transmission [3–5]. The infection spectrum of BTV is broad, ranging from subclinical infections to severe disease characterized by fever, edema, oral ulceration, respiratory distress, and lameness [6, 7]. Outcomes depend on host species [8], viral strain [9], and environmental conditions [10].

Numerous wild and domestic ruminants can serve as reservoirs for BTV, with serological surveys indicating widespread exposure even among clinically healthy individuals [11]. Since 1998, notifiable BTV Serotypes 1–4, 5, 6, 8, 9, 11, 12, 14, and 16 have been reported in Europe [12–16], with specific outbreaks exerting a particular impact on zoo species [17–19]. The 2006–2009 BTV‐8 outbreak showed that ruminants belonging to the subfamilies Bovinae and Caprinae were most severely affected, but also Cervidae and Camelidae could develop disease [17]. Even some nonartiodactyl and carnivore species were found seropositive, highlighting broad host exposure [11]. These events highlight the vulnerability of zoological collections, where diverse species living in close proximity face a high risk of infection and potential conservation and welfare consequences.

The resurgence of BTV highlights the ongoing risk posed by arboviruses in temperate regions, particularly in the context of climate change [20], which impacts the distribution, abundance, and seasonal activity of *Culicoides* vectors [21, 22]. In this context, BTV serotype 3 (BTV‐3) was first detected in Europe in Sicily in 2017 [23]. Although initially confined to the Mediterranean region, BTV‐3 emerged in the Netherlands in 2023, a strain distinct from the BTV‐3 strain found in Italy, which originated in Tunisia [13, 15]. Subsequently, the virus spread rapidly across Belgium and Northwestern Europe [3]. By summer 2024, explosive outbreaks of BTV‐3 were reported across Western Europe, affecting both livestock and wild ruminant species [3, 15, 24], including zoo‐housed animals [25]. In addition to BTV‐3, BTV‐12 was first detected in Europe in October 2024 in the Netherlands, bordering Belgium [16]. Currently, two distinct BTV‐8 strains are circulating in Europe: the strain that emerged in France in 2023, which is widespread and associated with clinical disease in ruminants [5]. The strain that reemerged in 2015 is only sporadically detected, primarily in animals without clinical signs [26]. Epizootic hemorrhagic disease virus serotype 8 (EHDV‐8), another *Culicoides*‐borne orbivirus, was first reported in mainland France, which borders Belgium, in September 2023 [5]. The temperature‐dependent ecology of *Culicoides* midges shapes BTV seasonality, with most clinical cases in Northwestern Europe occurring between July and December, and possible persistence through winter [17].

Since zoos are vulnerable to disease introduction and uniquely positioned for surveillance and conservation‐focused monitoring [27], studying BTV in these settings can offer valuable insights into host susceptibility and disease dynamics [17, 18, 28]. Furthermore, zoological collections house diverse, often endangered species for which the outcomes of BTV infection are poorly understood. Therefore, this study was designed to follow up on the impact of the BTV‐3 outbreak on two zoological collections in Belgium. Through the integration of systematic surveillance of viral presence and immune responses, clinical observations, and pathological examinations, this study seeks to advance understanding of host susceptibility and interspecies variability in clinical outcomes, and to inform strategies for the future management of potential BTV outbreaks in the conservation of vulnerable species. An extensive clinical and pathological characterization of the affected animals and species is beyond the scope of this article.

## 2. Materials and Methods

### 2.1. Study Population and Sampling

Between 2005 and 2025, 116 animals representing 33 species were sampled across two Belgian zoos (urban A and rural B). Zoo Antwerp (A) is located within a highly urbanized environment, characteristic of a dense city center. In contrast, Zoo Planckendael (B) is situated in a predominantly rural setting, embedded within a landscape of marshland, rivers, and streams. Serum samples collected prior to 2024 (retrospective) were obtained from our serum bank and stored at –20°C until transport and analysis. During the summer outbreak in August–September 2024 (outbreak), serum and EDTA whole blood were collected from clinically affected animals, including one European bison, three American bison, and four Damara goats. In November 2024, a postoutbreak screening campaign (follow‐up) was initiated to assess the extent of BTV‐3 exposure among zoo animals. Serum and EDTA whole blood samples were collected and stored at 8°C for no more than 72 h before transfer to the Belgian National Reference Laboratory (NRL, Sciensano). Follow‐up sampling was performed opportunistically during routine health programs, surgical procedures, clinical interventions, or medical examinations. Three American bison (*Bison bison*) and three guanacos (*Lama guanicoe*) were vaccinated against BTV‐8 in 2008; two mhorr gazelles (*Nanger dama mhorr*) against BTV‐3 in 2023 and BTV‐8 in 2022 in a Dutch and Spanish zoo, respectively. No other animals were vaccinated against BTV or epizootic hemorrhagic disease virus (EHDV) before the present study in our zoos. For each animal, epidemiological data were recorded, including species, sex, Zoological Information Management System (Species360) identification, sampling date, clinical status, and zoo of origin (A or B). Tables 1–4 outline the exact housing locations (Zoo A–B) of all animals, groups, and species at the time of sampling, thereby contextualizing the diagnostic assay results within their respective environments.

**Table 1:** Summary of the retrospective (pre‐2024) analysis of the species examined, number of individuals sampled, total serum samples analyzed, and positive detections differentiated by zoological institution (urban zoo—A; rural zoo—B) and taxonomic group across pan‐BTV ELISA, BTV‐3 VNT, and BTV‐8 VNT assays.

Taxonomic group	Zoo	Total species	Total animals	Serum samples	Positive pan‐BTV ELISA	Positive BTV‐3 VNT	Positive BTV‐8 VNT
Artiodactyla	A	2	2	2	0	0	0
	B	12	28	28	4	0	2
Proboscidea	A	1	3	3	0	0	0
	B	1	3	22	0	0	0
Perissodactyla	A	0	0	0	0	0	0
	B	5	8	14	4	0	0
Total		21	44	69	8	0	2

**Table 2:** Results of the acute outbreak and follow‐up screening of the species examined, number of individuals sampled, total serum and whole blood samples analyzed, and positive detections differentiated by zoological institution (urban zoo—A; rural zoo—B) and taxonomic group across pan‐BTV ELISA, BTV‐3 VNT, BTV‐8 VNT, and BTV‐3 PCR assays.

Taxonomic group	Zoo	Total species	Total animals	Serum samples	Whole blood samples	Positive pan‐BTV ELISA (%^a^)	Positive BTV‐3 VNT (%^a^)	Positive BTV‐8 VNT (%^a^)	Positive BTV‐3 PCR (%^a^)
Artiodactyla	A	7	12^b^	12	8	1 (9%^a^)	1 (9%^a^)	0	1 (13%^a^)
	B	15	59^b^	67	65	45 (78%^a^)	42 (72%^a^)	6 (10%^a^)	27 (47%^a^)
Proboscidea	A	1	1	2	1	0	0	0	0
Perissodactyla	B	4	6	8	4	3 (16%^a^)	0	0	0
Rodentia	B	2	3	3	3	0	0	0	0
Diprodontia	B	1	3	3	3	0	0	0	0
Carnivora	B	1	2	2	2	0	0	0	0
Total		31	86	97	86	49	44	6	28

^a^Proportion of ruminants testing positive, expressed as the percentage of positive individuals among all ruminants tested in each zoo.

^b^Total artiodactyls in urban Zoo A: 12 animals (11 ruminants and one warthog, *Phacochoerus africanus*); rural Zoo B: 59 animals (58 ruminants and one Chacoan peccary, *Catagonus wagneri*).

**Table 3:** Outbreak and follow‐up serological and molecular screening for bluetongue virus (BTV) in artiodactyl species from the two zoological collections: positive detections distributed across urban (Zoo A) and rural (Zoo B) settings.

Species	Zoo	Total animals	Positive pan‐BTV ELISA	Positive BTV‐3 VNT	Positive BTV‐8 VNT	Positive BTV‐3 PCR
European bison *Bison bonasus*	B	3	3 of 3 4–4	3 of 3 40–1280	1 of 3 40	3 of 3 23,19–34,52
American bison *Bison bison*	B	9	10/10^a^ 4–5	10/10 20 to >2560	4/10	10/10 26,07–40,00^b^
Watusi *Bos taurus watusi*	B	2	2/2 4–4	2/2 >2560 to >2560	0/2	2/2 33,31–33,31
Cape buffalo *Syncerus caffer*	A	1	1/1 4	1/1 640	0/1	1/1 38,28^b^
Eastern bongo *Tragelaphus eurycerus isaaci*	B	4	5/5^a^ 5–8	5/5 160–1280	0/5	1/5 36,46
Mhorr gazelle *Nanger dama mhorr*	B	7	6/9^a^ 5–39	4/6 20–640	0/6	2/9 33,05–36,00
Addax *Addax nasomaculatus*	B	3	2/3 4–4	2/2 160–640	0/2	1/3 34,23
Wapiti *Cervus canadensis canadensis*	B	4	4/4 4–11	4/4 40–160	0/4	4/4 34,14–35,34
Damara goat *Capra hircus damara*	B	8	4/8 4–35	3/4 20–1280	0/4	3/9 28,28–36,61
Barbary sheep *Ammotragus lervia*	B	4	5/5^a^ 4–19	5/5 80–2560	0/5	2/5 36,24–37,07
Guanaco *Lama guanicoe*	B	2	2/2 4–4	2/2 160–640	2/2 160 to >160	0/2
Vicugna *Vicugna vicugna*	B	4	3/4 4–7	3/3 1280 to >2560	0/3	0/4
Bactrian camel *Camelus bactrianus*	B	3	3/3 7–8	3/3 160–320	0/3	0/3

*Note:* Values in ELISA, virus neutralization test (VNT), PCR assays denote the minimum and maximum antibody titers and cycle threshold (CT) values, respectively, representing positive test outcomes. ELISA results ≥40 were considered negative, and <40 were considered positive. VNT results <1:20 were considered negative. PCR cycle threshold (CT) values of 1–38 were classified as positive, and CT values of >38 and <45 as doubtful and ≥45 as negative.

^a^One American bison, one Eastern bongo, one Mhorr gazelle, and one Barbary sheep were sampled more than once.

^b^Sample positivity is considered doubtful.

**Table 4:** Longitudinal monitoring of bluetongue virus (BTV) infection dynamics in individual animals based on ELISA, VNT‐3, VNT‐8, and PCR (BTV‐3) results.

Species	Specimen	Zoo	Month + year	Pan‐BTV ELISA	BTV‐3 VNT	BTV‐8 VNT	PCR BTV‐3
Indian rhino	1	B	September 2018	24	<20	<20	—
*Rhinoceros unicornis*			September 2022	38	<20	<20	—
			July 2023	21	<20	<20	—
			August 2023	20	<20	<20	—
			April 2024	10	<20	<20	—
			September 2024	19	<20	<20	45
			November 2024	20	<20	<20	45
European bison	1	B	March 2008	31	<20	<20	—
*Bison bonasus*			November 2024	4	1280	<20	34,52
	2	B	February 2011	118	—		—
			August 2024	4	40	<20	23,19
Eastern bongo	1	B	July 2023	104	—		—
*Tragelaphus eurycerus isaaci*			November 2024	5	1280	<20	40
			March 2025	5	Insufficient serum	Insufficient serum	45
American bison	1	B	February 2011	64	—	—	—
*Bison bison*			March 2014	118	—	—	—
			November 2024	4	1280	<20	38,18
	2	B	June 2012	93	—	—	—
			March 2014	95	—	—	—
			November 2024	4	80	<20	30,71
	3	B	March 2014	100	—	—	—
			August 2024	4	80	<20	35,77
	4	B	August 2015	108	—	—	—
			November 2024	4	1280	<20	35,57
	5	B	May 2016	111	—	—	—
			November 2024	4	>2560	<20	40
	6	B	August 2024	4	20	80	26,07
			January 2025	5	1280	160	34,01
Addax	1	B	August 2014	120	—	—	—
*Addax nasomaculatus*			November 2024	118	—	—	45
	2	B	July 2020	118	—	—	—
			November 2024	4	160	<20	45
Mhorr gazelle	1	B	September 2022	124	—	—	—
*Nanger dama mhorr*			November 2024	143	—	—	45
	2	B	August 2024	140	—	—	36
			November 2024	5	320	<20	33,05
			July 2025	4	640	<20	45
Damara goat	1	B	October 2022	89	—	—	—
*Capra hircus damara*			November 2024	90	—	—	45
Barbary sheep	1	B	December 2024	6	2560	<20	36,24
*Ammotragus lervia*			June 2025	4	2560	<20	45

*Note:* A notable incidence of BTV‐positive results was recorded during the outbreak (August–September 2024) and follow‐up screening in November 2024, highlighting clear viral activity. Numbers shown indicate positive test results. The symbol “—” denotes tests were not performed. ELISA results ≥40 were considered negative, and <40 were considered positive. VNT results < 1:20 were considered negative. PCR cycle threshold (CT) values of 1–38 were classified as positive, and CT values of >38 and <45 as doubtful and ≥45 as negative.

### 2.2. RNA Extraction and PCR

In Belgium, suspected clinical BTV cases are notifiable and subject to mandatory blood sampling. Samples were submitted to the NRL Sciensano. RT‐qPCR was performed to detect BTV and determine the serotype of clinical cases, while samples negative for BTV were subsequently screened for EHDV. RNA was extracted from EDTA blood samples using the Indimag 48 extraction robot with the Indimag Pathogen Kit (INDICAL BIOSCIENCE GmbH, Leipzig, Germany), following the manufacturer’s instructions.

BTV RNA was detected using a duplex RT‐qPCR assay combining a pan‐BTV reaction targeting segment 10 encoding the NS3 protein [29] with an endogenous control reaction targeting GAPDH, as previously described [3]. Amplifications were performed with the AgPath‐ID One‐Step RT‐PCR Kit (Thermo Fisher, Merelbeke, Belgium) using the following cycling conditions: 45°C for 10 min, 95°C for 10 min, followed by 45 cycles at 95°C for 15 s and 56°C for 45 s. Samples with Ct‐values ≤38 and showing exponential amplification curves were considered positive, whereas samples with Ct‐values >38 and <45 were classified as doubtful. Samples without amplification or with Ct‐values ≥45 were considered negative. A Ct < 35 for the endogenous control (GAPDH) was required to validate the PCR result.

Samples testing positive or doubtful in the pan‐BTV assay were further analyzed by serotype‐specific RT‐qPCRs targeting segment 2 encoding the outer‐capsid protein VP2. The BTV‐3‐specific assay followed the protocol of Lorusso et al. [30], and the BTV‐12‐specific assay used primers and probe described by Maan et al. [31]. Both assays employed the same amplification mix as for the pan‐BTV reaction, with an annealing temperature of 60°C.

For EHDV detection, a duplex RT‐qPCR assay combining a pan‐EHDV reaction targeting segment 9 encoding VP6 [32] with the same GAPDH control was applied, following identical cycling conditions.

### 2.3. ELISA

Antibody prevalence was determined by ELISA using a commercial pan‐BTV VP7 competition ELISA (ID SCREEN Bluetongue, Innovative Diagnostics, France) according to the manufacturer’s protocol. In short, serum samples and controls were incubated in antigen‐coated plates, allowing competition between antibodies in the sample and an HRP‐conjugated monoclonal anti‐VP7 antibody. After washing, substrate was added, and optical density (OD) was measured at 450 nm. Results were expressed as a competition percentage (S/N%), calculated as (OD sample/OD negative control) × 100. Samples with S/N% ≥ 40 were considered negative, and those with S/N% < 40 were considered positive.

### 2.4. Virus Neutralization Tests (VNTs)

The ELISA positive samples were confirmed by VNTs to determine the presence of serotype‐specific neutralizing antibodies. Distinction was made between Serotypes 3 and 8 using the BTV‐3 strain BEL 2024/01 and the BTV‐8 strain BEL 2006/02.

For the VNTs, serum samples were decomplemented for 30 min at 56°C. Twofold serial dilutions (1:10–1:2560) were prepared, and 50 µL of each dilution was incubated with 100 TCID_50_ of BTV‐3 for 1 h at 37°C. Subsequently, 50 µL of BHK cell suspension (2 × 10^4^ cells/well) was added. After 3 days of incubation at 37°C, cytopathic effect (CPE) was assessed microscopically. Neutralizing antibody titers were expressed as the reciprocal of the highest serum dilution preventing CPE in 50% of wells. Results of ≥1:20 were considered positive and <1:20 negative. Positive and negative controls were included in each assay.

### 2.5. Statistics

For the true ruminants (suborder Ruminantia), both ELISA results and VNT titers were log‐transformed and analyzed using the Kruskal–Wallis test with Bonferroni adjustment for multiple comparisons, with statistical significance set at *p* < 0.05 (IBM SPSS Statistics, Version 29). For VNT values exceeding 1:2560, an imputed titer of 1:5120 was assigned.

## 3. Results

### 3.1. Clinical BTV Cases and Outcomes

The first clinical signs during the outbreak occurred in August 2024, successively in European bison (*N* = 3/3, *Bison bonasus*), American bison (*N* = 7/9), Watusi (*N* = 2/2, *Bos taurus watusi*), Damara goat (*N* = 4/8, *Capra hircus damara*), and wapiti (*N* = 1/4, *Cervus canadensis canadensis*). Symptoms included lameness, conjunctival congestion, respiratory distress, oral ulcerations, and nasal discharge with substantial inter‐ and intraspecies variability. Ultimately, one European and one American bison died. Necropsy revealed suppurative laminitis, ulcerative stomatitis, multifocal subacute bronchitis, and myocardial necrosis. Morbidity and case fatality rates were 78% and 11% in American bison (*N* = 9) and 100% and 33% in European bison (*N* = 3), respectively.

### 3.2. Retrospective Serological Analysis

A total of five of 44 tested animals were seropositive by ELISA in the retrospective analysis of samples collected before 2024. One greater one‐horned (GOH) rhinoceros (*Rhinoceros unicornis*), one European bison, and one mhorr gazelle tested positive by ELISA but were negative in VNTs for BTV‐3 and BTV‐8. Another European bison and one guanaco tested positive by both ELISA and VNT‐8, but negative for VNT‐3 (Table 1).

### 3.3. Outbreak and Follow‐Up Screening

Since the outbreak, serological reactivity was confined to Artiodactyla and Perissodactyla. Uniformly negative results were obtained in the following taxa (Table 2): Proboscidea—Asian elephant (*N* = 1, *Elephas maximus*); Perissodactyla—Grévy’s zebra (*N* = 2, *Equus grevyi*), Przewalski’s horse (*N* = 1, *Equus caballus przewalskii*), and Somali wild ass (*N* = 2, *Equus africanus somaliensis*); Rodentia—Patagonian mara (*N* = 2, *Dolichotis patagonum*) and capybara (*N* = 1, *Hydrochoerus hydrochaeris*); Diprotodontia—swamp wallaby (*N* = 3, *Wallabia bicolor*); Carnivora—Asiatic lion (*N* = 2, *Panthera leo persica*). Across both zoos, 46 ruminants (67% of tested ruminants) and one GOH (16% of tested perissodactyls) tested positive in the ELISA (Table 2). For this ELISA, the test statistic across animal groups was 21.399, with an unadjusted *p*‐value of 0.018; after Bonferroni adjustment, the *p*‐value exceeded 0.05.

Neutralizing antibodies to BTV‐3 and BTV‐8 were subsequently detected in 43 (62%) and six (9%) ruminants, respectively. BTV‐3 viral RNA was identified in 28 tested animals overall, corresponding to 42% of tested ruminants, with no other orders yielding positive results (Table 2). The distribution across Zoo A and Zoo B is presented in Tables 2 and 3. For the VNT, the test statistic across animal groups was 17.137 (*p* = 0.071).

Negative artiodactyl results were obtained in the following species: rural zoo—giraffe (*N* = 3, *Giraffa camelopardalis antiquorum*), dorcas gazelle (*N* = 3, *Gazella dorcas*), and Chacoan peccary (*N* = 1, *Catagonus wagneri*); urban zoo—Kirk’s dik–dik (*N* = 1, *Madoqua kirkii*), red duiker (*N* = 1, *Cephalophus natalensis*), markhor (*N* = 4, *Capra falconeri*), alpaca (*N* = 3, *Vicugna pacos*), and warthog (*N = 1*, *Phacochoerus africanus*). Ruminant‐specific positive outcomes, including ranges of test values, are detailed in Table 3.

One European bison, three American bison, and two guanacos tested specifically positive for BTV‐8 by VNT. The mhorr gazelle vaccinated against BTV‐3 in 2023 tested ELISA positive but exhibited only a weak positive neutralizing antibody titer in the BTV‐3 VNT (1:20). In contrast, another mhorr gazelle vaccinated against BTV‐8 in 2022 was negative by both ELISA and VNT‐8.

### 3.4. Longitudinal Dynamics of Infection and Immunity

In total, 42 sampling events from 16 animals representing eight species were analyzed longitudinally, integrating pre‐ and post‐2024 results (Table 4). One GOH remained seropositive in ELISA, although VNT and PCR were negative. One Damara goat, one addax, and one mhorr gazelle remained negative in ELISA and PCR. A second addax became ELISA and VNT‐3 positive in the follow‐up screening, but remained negative in PCR and the BTV‐8 VNT. One pre‐2024 wisent sample tested positive in ELISA but remained negative in the VNT. The 11 other ruminants were serologically and PCR positive since 2024. One American bison remained RNA‐positive 5 months after the first detection, persisting into the vector‐free period.

### 3.5. BTV‐12 and EHDV

PCR analysis revealed no evidence of BTV‐12 or EHDV in any of the examined samples. All samples that were positive or doubtful in the pan‐BTV RT‐PCR were subsequently tested using the BTV‐3 serotype‐specific assay. Except for one sample, all pan‐BTV positive samples showed comparable Ct values in the BTV‐3 assay. One doubtful pan‐BTV sample could not be confirmed as BTV‐3; however, this sample displayed a high BTV‐3 VNT titer and was negative in the BTV‐8 VNT.

## 4. Discussion

This study was designed to follow‐up on the impact of the BTV‐3 outbreak on two Belgian zoological collections situated in distinct environments—one urban and one rural. The main findings of the study were: (i) high levels of seropositivity in ruminant species, (ii) variability in antibody response across taxa, (iii) prolonged persistence of viral RNA in selected individuals, and (iv) severe clinical impact of the disease on bison.

### 4.1. Comparative Susceptibility and Clinical Impact of BTV‐3, With Emphasis on Bison

The first major BTV‐8 epidemic in Northwestern Europe occurred between 2006 and 2010, spreading from the Netherlands to neighboring countries [12]. The current BTV‐3 epidemic originated from the Netherlands in 2023 [15, 23], and subsequently spread rapidly across the continent [24]. This strain differs from the BTV‐3 strain originally found in Italy in 2017 [13].

BTV‐8 susceptibility varies by host species, with sheep typically being more severely affected than cattle [33]. The circulating BTV‐3 strain from the current outbreak has been associated with more severe clinical manifestations than those observed in the earlier BTV‐8 outbreak in domestic sheep and cattle, and, to a lesser extent, in goats, ranging from subclinical infection to fatal disease [15, 24, 34]. American and European bison demonstrated marked susceptibility to BTV‐3 in our collection, with clinical signs in nearly all individuals and fatal outcomes observed in both species. The severity of necropsy findings, including laminitis, stomatitis, bronchitis, and myocardial necrosis, confirms the potential of BTV‐3 to cause substantial pathology in these taxa. Watusi cattle, wapiti, and Damara goats were affected to a lesser extent. The observed predominance of BTV‐3 infection among ruminants in our collection is consistent with earlier BTV outbreaks, in which the subfamilies bovinae and caprinae were most affected in European zoos [17, 19, 35]. During the 2006–2010 BTV‐8 outbreak in European zoos, affected Bovinae and Caprinae exhibited a case fatality rate of 69%. The American and European bison showed combined morbidity and case fatality rates of 37% and 50%, respectively [17, 35]. In contrast, our study found morbidity and case fatality rates due to BTV‐3 of 78% and 11% in American bison and 100% and 33% in European bison, respectively. In noncaptive environments, fatal cases of BTV‐3 in European bison have also been reported [36]. Based on our observations, we recommend targeted clinical and epidemiological surveillance of bison populations, particularly during outbreak periods.

### 4.2. Species‐Specific Diagnostic Patterns

Our data highlight clear species‐specific differences in BTV infection dynamics. ELISA results indicate prior exposure to BTV infection, VNT assays highlight variation in serotype‐specific neutralizing responses, and PCR demonstrates active viremia. Among true ruminants, ELISA and VNT results differed across taxa. For the ELISA, the Kruskal–Wallis test yielded a statistic of 21.399 with an unadjusted *p* = 0.018; however, differences were no longer significant after Bonferroni correction, likely due to small sample sizes in some groups. For the VNT, the Kruskal–Wallis test statistic was 17.137 (*p* = 0.071), indicating no significant differences even before adjustment, which may also be explained by the limited sample size.

#### 4.2.1. Retrospective Serological Findings

The retrospective serological screening performed on samples collected between 2005 and 2023 assessed BTV exposure prior to the current BTV‐3 outbreak (Table 1). One guanaco and one European bison tested seropositive for BTV‐8, consistent with previous vaccination in the guanaco and likely natural exposure to BTV‐8 in the European bison. Interestingly, the persistence of detectable antibodies and neutralization titers in the guanaco several years after vaccination suggests long‐lasting humoral immunity. Another European bison, one GOH, and one mhorr gazelle tested ELISA positive but were negative by VNT‐3 and VNT‐8, suggesting that the induced neutralizing antibodies diminished over time to undetectable levels or prior exposure to alternative serotypes not assessed in the assay. The latter, however, is less likely, as no BTV serotypes other than 3 and 8 have been detected in Belgium.

#### 4.2.2. Interspecies Differences in BTV3 Susceptibility Measured During and After the BTV‐3 Outbreak

Monitoring during the acute outbreak and subsequent months demonstrated broad exposure to BTV‐3 among ruminants (Table 2).

In Bovinae and Cervidae (wapiti), serological and molecular evidence of infection was nearly complete, with all individuals testing positive by ELISA, VNT‐3, and PCR, except for three bongo that were PCR negative (Table 3). These findings provide clear evidence of strong circulation of BTV‐3 and high susceptibility to BTV‐3 infection in these species. Additionally, three American bison and one European bison tested positive by VNT‐8. The three American bison had been vaccinated against BTV‐8 in 2008, indicating the persistence of detectable antibodies and neutralization titers several years after vaccination, suggesting long‐lasting humoral immunity. The European bison’s VNT‐8 positivity in the absence of vaccination or clinical signs suggests natural exposure and possible subclinical infection during the 2006–2010 BTV‐8 outbreak, similar to the other European bison in the retrospective analysis.

Among Caprinae (Table 3), two newly imported Barbary sheep from Germany were screened and found to be ELISA and VNT‐3 positive but PCR negative, indicating prior exposure with no remaining detectable viremia. This finding further suggests that BTV‐3 outbreaks had also occurred in Germany, underscoring the potential risk of introducing viremic animals into naive populations if asymptomatic carriers are not systematically tested. At the rural Zoo Planckendael, BTV‐3 infection was detected in Barbary sheep (*N* = 4/4) and Damara goats (*N* = 4/8). In contrast, all markhors (*N* = 0/4) housed in the urban Zoo Antwerp remained BTV‐3‐negative, likely reflecting lower infection pressure in the urban setting. Overall, Caprinae exhibited heterogeneous immune responses, ranging from strong serological ELISA reactivity to weak or absent VNTs. This pattern in our collection suggests that immunity in some caprine species may be short‐lived, potentially increasing susceptibility to reinfection and complicating long‐term surveillance and control efforts. Abera et al. [37] reported that goats in Ethiopia maintained high ELISA antibody titers following infection with an unspecified wild‐type BTV serotype. More research is needed to clarify the duration of seropositivity in goats within our collection.

Among Camelidae (Table 3), nearly 100% seropositivity was observed in all individuals from the rural zoo, except for one vicugna. In contrast, none of the alpacas (*N* = 3) housed in the urban zoo tested positive. No PCR positives were identified during the postoutbreak screening, suggesting that Camelidae may clear viremia efficiently. These findings indicate that while Camelidae are susceptible to BTV‐3, their role in maintaining active infection within our collection appeared limited. This observation is consistent with the earlier BTV‐8 outbreak, where occasional cases were reported [17, 38, 39], but South American camelids were found to play only a negligible role in the epidemiology of the virus [40]. In another study, the same authors performed an infection trial of BTV‐8 in three alpacas and three llamas (*Lama glama*). This experimental study demonstrated low seroreactivity and delayed, weak antibody responses to BTV‐8. Viral RNA levels in blood were minimal and rapidly cleared, indicating very limited susceptibility to sustained viremia [41]. In our study, two guanacos tested positive for BTV‐8 VNT in the postoutbreak screening. These results are consistent with previous vaccination, indicating long‐lasting humoral immunity in these animals. Within Antilopinae, only mhorr gazelles (*N* = 7) showed evidence of infection, with five testing positive by ELISA, three by VNT‐3, and one by PCR, indicating a heterogeneous immune response. The mhorr gazelle vaccinated against BTV‐3 in 2023 demonstrated a weak VNT‐3 titer, whereas another vaccinated against BTV‐8 in 2022 was negative. The findings suggest a potentially short‐lived immune response, implying that frequent booster vaccinations may be required to sustain sufficiently high antibody titers for effective protection. The remaining Antilopinae (Dorcas gazelles, *N* = 3, rural zoo; Kirk’s dik‐dik, *N* = 1, urban zoo) and the Cephalophinae (red duiker, *N* = 1, urban zoo) were all BTV‐3 negative. These results suggest that these species were either not exposed during the outbreak or exhibit a very limited susceptibility to BTV‐3 under the observed conditions. Bluetongue has circulated in sub‐Saharan wildlife for centuries [42], with experimental infections in blesbuck (*Damaliscus albifrons*) first demonstrating subclinical disease yet sufficient viremia to infect sheep [43]. Later surveys confirmed widespread exposure, with antibodies detected in buffalo, wildebeest, elephants, giraffes, and other species, underscoring the role of African wild ruminants as reservoirs without overt disease [17, 44].

In this study, viral RNA was detected exclusively in ruminants, indicating active replication with potential for onward transmission via *Culicoides* vectors. By mid‐September 2024, Belgian authorities suspended confirmatory testing as BTV‐3 had already been confirmed in nearly 3000 herds nationwide [3]. The PCR positivity observed in the November–December follow‐up screening likely underestimated the percentage of viremic animals during and immediately after the acute outbreak, as some animals had already cleared viremia. This is underscored by the higher percentage of seropositive ruminants. Cattle may remain PCR positive for BTV nucleic acid for 6 months or longer after infection, a persistence that complicates interpretation, as RT‐qPCR alone does not always confirm active virus circulation or the presence of infectious virus [7]. This prolonged detectability of nucleic acids has significant implications for conservation management, as it can impede the international transport of valuable individuals and thereby hinder the progress of coordinated breeding programs for threatened species.

Cattle species and, to a lesser extent, goats are generally regarded as important amplifying hosts of BTV, as they can sustain prolonged viremia in the absence of overt clinical signs [45–47]. Amplifying hosts play a critical role in virus transmission and in shaping the epidemiology of BTV in naive populations [45]. However, in our study, goats displayed heterogeneous and weak immune responses and PCR positivity, suggesting a more limited contribution to virus maintenance for the BTV‐3 strain in the 2024 outbreak.

#### 4.2.3. Longitudinal Insights Into Infection and Immunity

Longitudinal monitoring of affected individuals offers valuable insight into the durability of immune responses as well as the potential persistence of viremia, maintenance of virus beyond the active vector period, and patterns of resurgence. One American bison demonstrated prolonged detectability of viral RNA for up to 5 months, illustrating species‐specific variation in postinfection dynamics (Table 4). The GOH rhinoceros was consistently ELISA positive, including in 2022, before BTV‐3 was detected in Western Europe. This discrepancy may indicate false positives, an absence of robust serotype‐specific neutralizing antibodies, or exposure to other serotypes not tested. As the assays used were not validated for rhinoceros samples, some degree of cross‐reactivity or nonspecificity cannot be excluded. This highlights the importance of interpreting ELISA, VNT, and PCR results together, as each method captures distinct aspects of infection dynamics and the immune response.

Knowledge on the duration of immunity after BTV vaccination with inactivated vaccines is limited in domestic ruminants [11], and even scarcer in wild species. In our collection, weak or absent antibody responses in previously vaccinated mhorr gazelles underscore the need for longitudinal postvaccination screening to come up with recommendations. Evidence of reduced immunity should guide vaccination strategies, particularly for animals that are therefore at heightened risk of reinfection.

#### 4.2.4. Infection Patterns in the Urban and Rural Zoos

Zoo Antwerp (A) is located within a highly urbanized environment, characteristic of a dense city center. In contrast, Zoo Planckendael (B) is situated in a predominantly rural setting, embedded within a landscape of marshland, rivers, and streams. Only a single Cape buffalo in the urban collection of Zoo Antwerp tested positive for BTV‐3 (Tables 2 and 3). In contrast, the rural collection at Zoo Planckendael, where a substantially larger number of ruminants were sampled, showed widespread infection across multiple species. This difference likely reflects variation in infection pressure between settings, with rural environments, particularly those near wetlands, posing a greater risk of BTV transmission due to higher vector abundance and increased host density associated with surrounding agricultural livestock. These findings highlight the importance of considering the ecological context when interpreting surveillance data from zoological collections.

The higher infection pressure in the rural collection at Zoo Planckendael is likely explained by local vector ecology. *Culicoides* midges develop in wet, organic‐rich habitats such as floodplains, wetlands, and agricultural dung substrates [48], all of which are more abundant in rural settings. Given *Culicoides*’ limited flight range of only a few kilometers [49], transmission risk is strongly influenced by proximity to suitable breeding sites, vicinity of other suitable hosts, and climatic conditions supporting dense vector populations [50]. By contrast, the urban setting around Zoo Antwerp provides few suitable habitats for *Culicoides*, and neighboring farms with susceptible hosts are located several kilometers away. These factors likely contributed to the markedly lower level of BTV circulation there.

#### 4.2.5. Future Directions and Limitations

Mitigating the threat of emerging BTV serotypes and other vector‐borne diseases such as EHDV in zoological collections will depend on continuous longitudinal monitoring, improved vector surveillance under shifting climatic conditions, and tailored vaccination strategies for at‐risk species. Crucially, incorporating zoo‐derived data into regional and European surveillance programs could strengthen early warning systems and help safeguard both animal health and conservation breeding efforts.

This study relied on opportunistic sampling, which limited systematic comparisons between taxa and across time points. Sample sizes for several species were small, and serological interpretation is subject to the inherent limitations of ELISA and VNT assays regarding sensitivity and specificity. Nonetheless, the longitudinal approach, integrating samples collected before, during, and after the 2024 outbreak, provided valuable insights into infection dynamics across a diverse range of species.

## 5. Conclusion

This study provides the first comprehensive overview of a BTV‐3 outbreak in two Belgian zoos, demonstrating high susceptibility and severe clinical outcomes in bison, broad exposure across ruminant taxa, and marked interspecies differences in infection dynamics and immune responses. The absence of BTV‐12 and EHDV highlights the specificity of the outbreak, while epidemiological contrasts between urban and rural settings underscore the influence of local vector ecology. These findings emphasize the value of zoological collections as sentinels for emerging arboviruses and underline the need for sustained surveillance, tailored vaccination strategies, and integration of zoo‐based data into wider disease control and conservation frameworks.

## Funding

The Centre for Research and Conservation receives structural support from the Flemish Government.

## Ethics Statement

All sample collection was performed by trained zoo staff. Samples were either retrieved from the existing serum bank or obtained opportunistically from animals undergoing routine medical programs, health check‐ups, or surgical interventions during the study period. As no procedures were performed solely for research purposes, formal ethical approval was not required.

## Conflicts of Interest

The authors declare no conflicts of interest.

## Data Availability

The data that support the findings of this study are available from the corresponding author upon reasonable request.
